# Dental Education

**Published:** 1853-04

**Authors:** 


*Dental Education.*—Our friend, Dr. Wood, of the "Southern Journal of the Medical and Physical Sciences," seems to think we did him injustice in our remarks in the January number of our journal. We had no such intention. We merely stated from recollection what we conceived to be the general purport of his several articles on the subject of dental education. We would not willingly misrepresent any one. In opposing the several schemes of dental education which have recently been submitted to the consideration of the profession, we have been influenced by the conscientious belief that they were either impracticable or would, if carried out, have the effect of lowering the standard of qualification, and we are happy to know, too, that this opinion is entertained by most of the best educated dentists in the United States. Indeed, we have received letters from dentists, standing high in the profession, from almost every part of the country commending the course which we have pursued in relation to this subject.

We have no objection to one nor to half a dozen preliminary courses in Medical Colleges, but we do not think that Dental Colleges have any more right to require it as a condition of matriculation than the former institutions to demand a preliminary course in the latter. We have always advocated the importance of a knowledge of general medicine to the practitioner of dental surgery; and dental colleges, as at present constituted, are provided with the most ample means for giving as thorough instruction in all the branches of medical science which it is really necessary for the dentist to understand, as any medical college. If some of the gentlemen who entertain so humble an opinion of the amount of medical instruction given in the schools of dentistry would attend the lectures from the chairs of these departments one session, their views upon the subject doubtless would be very greatly enlarged.

